# Impact of Family-Centered Early Intervention in Infants with Autism Spectrum Disorder: A Single-Subject Design

**DOI:** 10.1155/2020/1427169

**Published:** 2020-03-03

**Authors:** Ho Il Park, Hae Yean Park, Eunyoung Yoo, Areum Han

**Affiliations:** ^1^Department of Occupational Therapy, Seoul Metropolitan Children's Hospital, Republic of Korea; ^2^Department of Occupational Therapy, Yonsei University, Republic of Korea

## Abstract

**Objective:**

Autism spectrum disorder is a neurodevelopmental disorder that affects communication, social skills, and behavior and can present in early childhood. The present study is aimed at investigating the effects of family-centered early intervention on the quality of social interaction and social interaction skills in infants with suspected autism spectrum disorder using a single-subject design.

**Method:**

As a single-subject design study, evaluations were conducted at baseline phase A, intervention phase B, baseline phase A′, and follow-up phase. The family-centered early intervention program was implemented during the intervention phase. Family-centered early intervention programs included home environmental modification, play video recording and training, task and feedback, related information training, and question and answer. The Modified Checklist for Autism in Toddlers, Revised, with Follow-Up and Evaluation of Social Interaction was used for evaluation.

**Result:**

Three participants completed the study. After applying the family-centered early intervention program, the quality of social interaction and social interaction skills of all participants improved. In addition, the risk of autism spectrum disorder in all participants was reduced.

**Conclusion:**

Family-centered early intervention was confirmed to have a positive effect on the improvement of social interaction skills in infants with suspected autism spectrum disorder.

## 1. Introduction

Autism spectrum disorder (ASD) is a neurodevelopmental disorder that affects communication, social skills, and behavior and can present in early childhood [1]. Children with ASD have difficulties in forming relationships with others and are indifferent to social stimuli [2]. Social interaction and communication are the most important challenges in children with ASD [3].

Symptoms of ASD usually appear within the first 2 years of life [1]. Early intervention before 3 years old indicates a positive prognosis for the development of social interaction and communication skills [4]. Early intervention promotes complex neural networks and connectivity [5] along with brain development through synaptic overproduction [6]. Therefore, early intervention in early childhood, when neuroplasticity is most active, can improve social interaction and communication functions in those with ASD [7].

Effective early intervention to improve social interaction and communication skills of infants with ASD is a family-centered approach based on play [8, 9], which has a positive impact on social interaction and communication [10]. Children can interact with the environment through play and learn socialization, social interaction, communication, and creativity [11]. The caregiver is the first playmate in the infant's life, and the infant forms a positive affectional bond with others through play. This promotes the development of an infant's social interaction skills [12] and is the basis for participation in other environments [13].

The results of family-centered early intervention studies on ASD showed that social interaction skills were improved. The effects of early intervention through parent education included reduced parent stress, improved self-efficacy, and higher satisfaction and improvement of infants' social interaction skills [13, 14].

When adopting family-centered early intervention, each infant with ASD requires a suitable approach for their condition. Individuals with ASD have unique characteristics, which must be identified for implementation of a multidisciplinary approach. The occupational therapist is a key figure in the evaluation and intervention process of successfully treating child social impairments [15, 16]. The Occupational Therapy Intervention Process Model (OTIPM) is an inference method based on a multidimensional approach that can be used by clinicians with client-centered, occupation-based, and occupation-centered approaches [17]. When using OTIPM, identifying the client-centered context and selecting and applying individualized intervention methods are possible [18].

As previously mentioned, early intervention is needed to improve social interaction skills of infants with ASDs. Hence, this study is aimed at investigating whether individualized family-centered early intervention is effective in improving the social interaction skills of infants aged below 3 years with suspected ASD using a single-subject design.

## 2. Methods

### 2.1. Participants

The study participants were three infants with suspected ASD. Infants (1) with a score of ≥8 in the Modified Checklist for Autism in Toddlers, Revised, with Follow-Up (M-CHAT-R/F) [19]; (2) aged between 24 and 36 months; (3) not diagnosed with disorders other than ASD; and (4) whose caregivers agreed to participate in the study were included.

### 2.2. Research Design

In this study, the ABA design was used for the single participants and included participant recruitment, baseline phase A, intervention phase B, baseline phase A′, and follow-up phase [20]. The study period was approximately 14 weeks, ranging from January 7, 2018, to April 15, 2018, after the institutional review board's approval date.

## 3. Measures

### 3.1. Modified Checklist for Autism in Toddlers, Revised, with Follow-Up

The M-CHAT-R/F was used as a tool to evaluate the screening and risk changes of infants with ASD. M-CHAT-R/F is a screening tool used for risk assessment and follow-up of ASD in infants aged between 16 and 30 months. The total score ranged from 0 to 20 points; the participants were classified into the low-risk group (0–2 points), the middle-risk group (3–7 points), and the high-risk group (8–20 points) (Robins et al., 2009). For the high-risk group, early intervention should be immediately performed. The internal consistency of the test (Cronbach's *α*) was 0.79 [21].

### 3.2. Evaluation of Social Interaction Second Edition

The Evaluation of Social Interaction (ESI) was designed to assess a person's quality of social interaction as he or she engages in “real” interactions. The ESI provides a standardized measure of a person's quality of social interaction as observed in a natural context to (a) establish a baseline level of performance, (b) plan occupational therapy services, and (c) measure progress or change over time, including the effectiveness of occupational therapy services. The reliability of the test was 0.87, and the internal consistency (Cronbach's *α*) was 0.94 [22].

## 4. Procedures

### 4.1. Participant Recruitment

Recruitment, selective assessment, and preevaluation were performed during the participant recruitment phase. The advertisement for study participants was posted in an online community (developmental delay parent meeting site). The applicants were visited at home to explain the research and to conduct screening evaluations. In the screening evaluation, applicants who received an M-CHAT-R/F score of >8 were preevaluated. ESI, Denver Developmental Screening Test-II, Revised Knox Preschool Play Scale, and Social Maturity Scale were used in the preevaluation.

### 4.2. Baseline Phase A

Baseline phase A was conducted three times a week. The researchers visited each applicant's home, watched the free play between the caregiver and the participant, and recorded a video to observe the quality of social interaction. When the caregiver and the participant naturally began to play freely, the researchers started to film. After 10 minutes, the recording was stopped, and the free play was ended.

### 4.3. Intervention Phase B

Intervention phase B was conducted 12 times over 6 weeks after the end of baseline phase A. For the experiment, the researcher visited the participant's home and provided parent education consisting of analysis of photographed images, revision, and suggestion of a play method beneficial for improving social interaction skills and presentation of tasks to improve social interaction skills. Tasks were performed daily, and their responses were recorded in the task notes. The researcher provided feedback to the parents at every session. The assigned researcher contacted the caregiver through daily messages to confirm the performance of the assignment.

### 4.4. Baseline Phase A′

Baseline phase A′ was performed three times a week after the intervention phase. The researcher visited the participant's home and photographed the free play situation of the participant and the caregiver. In the last session, the subject of the intervention was discussed.

### 4.5. Follow-Up Phase

To investigate the effect of family-centered early intervention on the social interaction of infants with suspected ASD, follow-up tests were performed 4 weeks after the end of baseline phase A′. Follow-up tests were conducted with one session of free play, M-CHAT-R/F, and ESI.

## 5. Independent Variable (Family-Centered Early Intervention Program)

This study used a family-centered early intervention program to improve the social interaction skills of infants with suspected ASD. Family-centered early intervention programs included home environment modification, play video recording and training, task and feedback, related information training, and questions and answers.

### 5.1. Home Environmental Modification

In this study, environmental modification was performed to improve the quality of social interaction of the participants. The environmental modifications applied in this study were teaching changes in household structure and providing and using applicable sensory tools. The most common method to modify the environment was video media restrictions, reducing unnecessary visual and auditory stimuli, providing individual space, and vestibular and proprioceptive stimuli [23].

### 5.2. Play Video Recording and Training

In this study, video recording was conducted to measure the frequency of social interaction techniques in the free play situation with the caregiver. The progress of the participant based on video recording was revised and supplemented according to the study of Bakeman and Adamson [24]. The free play took place in the participant's home and involved toys provided to the participant. In this study, toys included balls, picture books, puzzles, telephones, dolls, cars, and other items of interest [25]. The caregiver induced social interaction skills (looks, gesticulates, and produces speech) [22].

The researcher educated the caregiver to induce play according to the target level and recorded a video of the free play for 10 minutes. The researcher analyzed the recorded images at each session and educated the caregiver based on the analyzed contents.

### 5.3. Task and Feedback

In this study, we selected and applied the necessary task methods for each participant by referring to the OTIPM. The researcher interviewed a caregiver on the performance context and analyzed the difficulties of social interaction skills based on the performance context and evaluation. Subsequently, the causes affecting the participant's behavior were analyzed, and the tasks were applied based on the analyzed results [17].

### 5.4. Related Information Training

The researcher provided and educated the caregiver with information related to the subject. The training provided the necessary information to the individual by referring to the OTIPM.

For example, information provided mainly included parenting methods, joint attention, applied behavior analysis, DIR/floortime, and communication enhancement strategies [13, 26].

### 5.5. Task Fulfillment Rate

The researchers analyzed the task fulfillment rate of the caregivers by referring to the task notes. The analyzed task fulfillment rates were 81%, 100%, and 95% for participants 1, 2, and 3, respectively.

## 6. Dependent Variable

### 6.1. Session-Dependent Variable (Frequency of Social Interaction Skill Change)

The social interaction skills measured in this study were looks, gesticulates, and produces speech. Based on the definitions used in Fisher and Griswold [22], we operationally defined these three social interaction skills. To determine the frequency of social interaction skills used by the participant, the free play situation was recorded for 10 minutes at each session, and the researcher analyzed the recorded images. The frequency of social interaction skills was recorded, and behavioral analysis was conducted by two occupational therapists with 9 years of clinical practice.

The operational definitions of the social interaction skills are listed in Table 1.

### 6.2. Pre- and Postintervention-Dependent Variables

#### 6.2.1. Changes in the Quality of Social Interaction

We measured the quality of social interaction using the ESI. The intended purpose of these social interactions is interacting with others in the context of and in relation to a game (CP-3) and engaging in “small talk” with others while playing together (CS-4) [22]. Results were expressed as logit scores converted using Occupational Therapy Assessment Package software.

#### 6.2.2. Change in the Risk of Autism Spectrum Disorder (ASD)

The risk of ASD was measured using M-CHAT-R/F.

## 7. Analysis

The frequency of social interaction techniques during free play was recorded at each session and visually analyzed using linear graphs and 2 standard deviation (2SD) bands. In the pre-, post-, and follow-up phases, ESI and M-CHAT-R/F results were assessed and compared with bar graphs to indicate changes in the quality of social interaction and the risk of ASD.

The results of the social interaction skills in the play situation were analyzed by a researcher. To improve reliability, two pediatric occupational therapists and a researcher conducted preliminary training until the interobserver reliability was >90%. The interobserver reliability was calculated based on frequency. In this study, one-third of all images were randomly selected for each participant; the interobserver reliabilities for participants 1, 2, and 3 were 90%, 91%, and 93%, respectively.

## 8. Results

### 8.1. Participants

Three participants completed the study, and the general characteristics of the participants are described in Table 2.

## 9. Changes after the Intervention Sessions

### 9.1. Changes in Look Frequency

Participant 1 showed an increase of 273% from the mean 3.0 (range, 0–5) during baseline phase A to 8.2 (range, 0–18) after the intervention. Participant 2 showed an increase of 662% from 1.6 (range, 1–3) during baseline phase A to 10.6 (range, 4–20) during the intervention phase. Participant 3 showed an increase of 332% from 5.3 (range, 4–7) during baseline phase A to 17.6 (range, 7–25) during the intervention phase.

**Figure 1:**
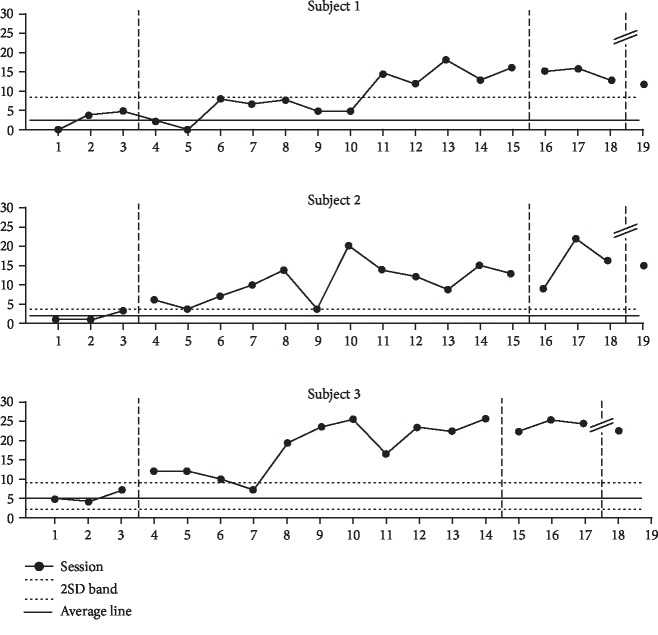
Changes in look frequency.

The significance of the intervention effect was confirmed by the 2SD band method, and the intervention results of all participants were continuously deviated over the 2SD band. The visual analysis is shown in Figure 1.

### 9.2. Changes in Gesticulate Frequency

Participant 1 showed an increase of 307% from 2.6 (range, 1–4) during baseline phase A to 8.0 (range, 0–15) during the intervention phase. Participant 2 showed an increase of 900% from 1.3 (range, 1–2) during baseline phase A to 11.7 (range, 1–28) during the intervention phase. Participant 3 showed an increase of 293% from 8.0 (range, 6–10) during baseline phase A to 23.5 (range, 12–30) during the intervention phase.

**Figure 2:**
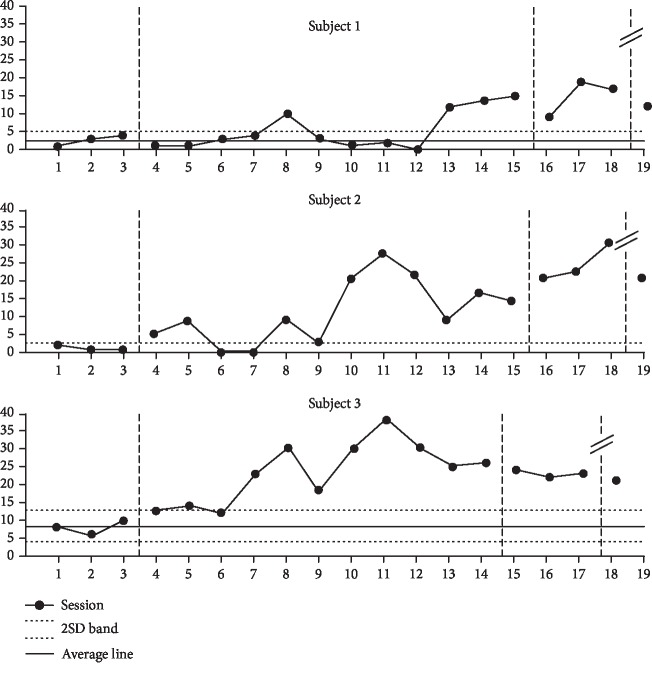
Changes in gesticulate frequency.

The visual analysis is shown in Figure 2.

### 9.3. Changes in the Frequency of Produced Speech

Participant 1 showed an increase of 477% from 5.3 (range, 4–6) during baseline phase A to 25.3 (range, 8–38) during the intervention phase. Participant 2 did not show changes between baseline phase A and the intervention phase. Participant 3 showed an increase of 545% from 3.3 (range, 3–4) during baseline phase A to 18.0 (range, 6–30) during the intervention phase.

**Figure 3:**
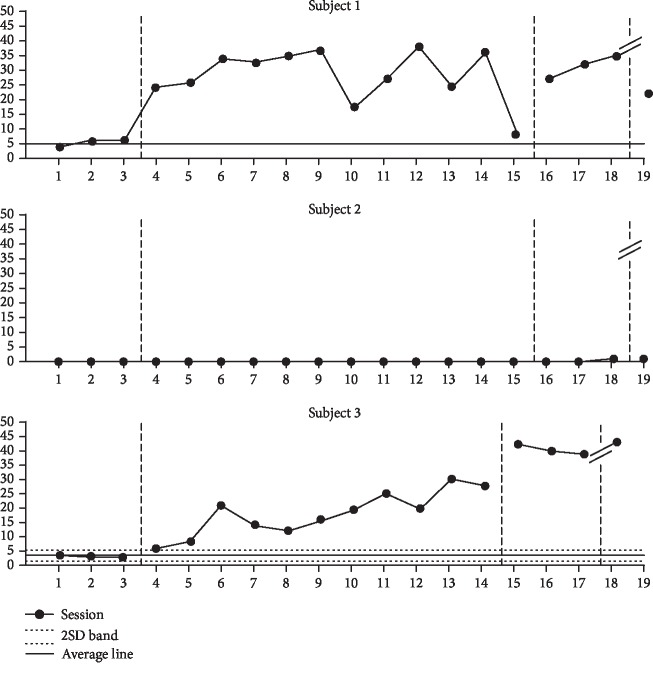
Changes in frequency of produced speech.

The intervention results of participants 1 and 3 continuously deviated over the 2SD band. The visual analysis is shown in Figure 3.

## 10. Changes in Pre-, Post-, and Follow-Up Phases

### 10.1. Changes in Quality of Social Interaction

The ESI logit of participant 1 increased to 0.4 logit (−1.8 to −1.4), and the most noticeable changes were turns toward, looks, produces speech, regulates, and replies. The ESI logit of participant 2 increased to 0.2 logit (−1.7 to −1.5), and the most noticeable changes were turns toward, looks, and gesticulates. The ESI logit of participant 3 increased to 0.6 logit (−2.0 to −1.4), and the most noticeable changes were turns toward, looks, gesticulates, produces speech, regulates, questions, and replies.

### 10.2. Changes in the Risk of ASD

The M-CHAT-R/F score of participant 1 was 8 points (high risk), and the postscore changed to 4 points (middle risk). The score of participant 2 was 11 points (high risk), and the postscore changed to 4 points (middle risk). The score of participant 3 was 8 points (high risk), and the postscore changed to 0 points (low risk).

## 11. Discussion

This study is aimed at investigating the effects of individualized family-centered early intervention on social interaction skills and the risk of ASD in infants with suspected ASD. We observed some changes in social interaction skills through video recording and analyzing the free play and quality of social interaction in play situations before and after the intervention and examined whether the effects persisted.

After applying the family-centered early intervention program, all participants significantly improved during the intervention phase and continuously improved after the intervention. In particular, participants 2 and 3 had little interaction with the caregiver before the intervention, but they used and learned to use social response, social smile, and gestures in the free play situation during the intervention. In participant 1, produced speech was significantly improved from the early stage of intervention. Participant 3 was only able to speak the word mother during baseline phase A, but the infant began to speak in sentences during intervention. This result is similar to the study of Wong and Kwan [27], showing improved social interactions and communication functions for eye contact, gesture, and vocalization/words as a result of early intervention in children with ASD younger than 36 months.

A task note was provided to confirm the task performance of the participants, and the caregiver recorded the performance of the task based on the session and the reactions of the participant. Based on the task notes, the parental performance of the caregivers improved, and the internal motivation for participating in the research could be improved through feedback. Additionally, the researcher was informed about the task notes in advance.

The ESI score was expressed as logit, and a change greater than 0.3 logit was interpreted as being clinically meaningful. According to the ESI evaluation result, logit scores improved by 0.5, 0.2, and 0.9 for participants 1, 2, and 3, respectively. The abilities to turn toward, look, and gesticulate were improved in all participants, and the change in participant 3 was significant. Many studies also say that family-centered early intervention improves social interaction skills [8, 9, 12, 27].

Accurately diagnosing ASD in infants under the age of 3 years is difficult, and their characteristics may change rapidly; thus, monitoring the risk of ASD is necessary. The M-CHAT-R/F allows screening for the risk of ASD before age 3 [21]. The changes in the M-CHAT-R/F results showed improvement in all participants. In particular, all items relating to joint attention, eye contact, and social smile were changed in participant 3, and the postevaluation result of M-CHAT-R/F was evaluated as score 0, which means the risk of ASD was eliminated through family-centered early intervention. In the present study, family-centered early intervention appeared to be effective in enhancing social interactions and communication functions of participants with ASD.

## 12. Limitations and Future Research

According to the result of this study, participant 3 showed the biggest improvement in all evaluations. We have confirmed that social interaction skill ability, ESI logit, and M-CHAT-R/F score were correlated; however, it is also necessary to study the correlation between each evaluation because of the small number of cases.

## 13. Conclusion

Three important results were obtained in this study.

First, the social interaction skills of all participants improved. All participants showed significant changes in looks, gesticulates, and produced speech, all of which were improved and maintained after the intervention was complete.

Second, the quality of social interaction of all participants was improved. The results of the ESI evaluation showed significant changes in skills such as turns toward, looks, gesticulates, produces speech, regulates, questions, and replies.

Third, the score of M-CHAT-R/F significantly decreased in the participants with ASD.

## Figures and Tables

**Table 1:** Operational definitions of social interaction skills.

Social interaction skills	Operational definition
Looks	Looking for more than 2 seconds (e.g., eye contact)
Gesticulations	Using gestures for communication (e.g., hand movement and smile)
Produced speech	Using words to communicate

**Table 2:** General characteristics of the participants.

	Participant 1	Participant 2	Participant 3
Gender	Male	Male	Male
Age	29 m	25 m	24 m
M-CHAT-R/F	8 points	11 points	8 points
ESI			
Logit	−1.8 logit	−1.7 logit	−2.0 logit
Percentile rating	9.5%	15.8%	3.5%
RKPPS	21.75 m	17.25 m	19.5 m
SMS			
Social age	21 m	19 m	18 m
Social quotient	78	82.38	69.16
DDST-II			
Personal-social	20 m	15 m	18 m
Fine motor-adaptive	33 m	24 m	22 m
Language	19 m	9 m	16 m
Gross motor	18 m	18 m	20 m

M-CHAT-R/F: Modified Checklist for Autism in Toddlers, Revised, with Follow-Up; ESI: Evaluation of Social Interaction; RKPPS: Revised Knox Preschool Play Scale; SMS: Social Maturity Scale; DDST-II: Denver Developmental Screening Test-II.

## Data Availability

The data used to support the findings of this study are available from the corresponding author upon request.
